# Aortic Thrombus: A Rare Cause of Ischemic Stroke

**DOI:** 10.7759/cureus.115201

**Published:** 2026-08-25

**Authors:** Khadija Ouchen, Naima Chtaou, Siham Bouchal, Youssef Alaoui Lamrani, Aouatef El Midaoui, Mustapha Maaroufi, Faouzi Belahsen

**Affiliations:** 1 Neurology, Hassan II University Hospital Centre, Fez, MAR; 2 Laboratory of Epidemiology and Research in Health Sciences, Faculty of Medicine, Pharmacy, and Dentistry of Fez, Sidi Mohamed Ben Abdellah University, Fez, MAR; 3 Radiology, Hassan II University Hospital Centre, Fez, MAR

**Keywords:** aorta, aortic arch, etiology, ischemic stroke, thrombus

## Abstract

Aortic thrombus is a rare and relatively underrecognized cause of peripheral embolic events, particularly ischemic stroke. It affects the elderly in most cases. It’s a retrospective case series study carried out in the Neurology Department of the Hassan II University Hospital of Fez, Morocco, to analyze the epidemiological, clinical, therapeutic, and outcome data of aortic thrombus as an etiology of ischemic stroke. Eight patients were included in this study, with a mean age of 65.87 years. They were admitted with an average delay of 12 hours and 56 minutes from the onset of the neurological symptoms. The imaging protocol consisted of a non-contrast CT scan combined with CT Angiography of the polygon of Willis and supra-aortic trunks. The aortic arch was the predominant site of thrombus. Treatment consisted of curative anticoagulation for all patients, whose continuation depends on the results of control imaging. The main etiology of aortic thrombus is represented by atherosclerosis, alongside other etiologies such as thrombophilia, infectious aortitis, particularly syphilitic aortitis and antiphospholipid syndrome. The main site is the transverse thoracic aorta and the isthmus. The optimal therapeutic approach remains controversial due to the lack of large prospective series in the literature and largely relies on curative anticoagulation. The use of thrombolysis is debated due to the risk of induced peripheral emboli. Thrombectomy is reserved for specific cases.

## Introduction

Stroke represents a major public health burden worldwide [1]. Considerable progress has recently been made in the management of ischemic stroke, and a more systematic etiological workup has proven essential, as identifying the underlying mechanism directly determines the choice of secondary prevention and thereby reduces the risk of recurrence. Nevertheless, approximately 25% of ischemic strokes remain cryptogenic [2]. The increasing use of computed tomography angiography (CTA) in the etiological assessment has progressively led to the identification of less common structural causes of ischemic stroke, such as aortic thrombus.

Aortic thrombus is defined as the presence of a thrombus in one or more segments of the aorta, regardless of its anatomopathological nature. It is a rare and relatively underrecognized cause of peripheral embolic events, including ischemic stroke. It predominantly affects elderly patients, most often as a complication of atherosclerosis, but can also occur in young adults in the setting of infectious or systemic disease. Although rare, ischemic stroke secondary to aortic thrombus has been increasingly documented in the literature, with reports highlighting the associated risk factors, clinical profiles, and therapeutic management [3-6].

We report a retrospective case series of eight patients admitted to the Neurology Department of Hassan II University Hospital of Fez, Morocco, for ischemic stroke, in whom the etiological workup identified an aortic thrombus as the most probable cause. Our study aimed to describe the epidemiological characteristics (age, sex, and vascular risk factors), clinical and radiological profiles, therapeutic management, and outcomes of these patients. We also report our experience with direct oral anticoagulants (DOAC) in the management of aortic thrombus.

## Materials and methods

Conception and study setting

This is a retrospective observational case series carried out in the Neurology Department of Hassan II University Hospital of Fez, Morocco, over a 45-month period from February 2020 to November 2023, including eight patients, with the contribution of the Radiology Department.

Study population

The study included all patients with recent ischemic stroke or transient ischemic attack diagnosed on non-contrast CT scan, whose etiological workup revealed an aortic thrombus on CTA, either as an emergency in the context of a thrombolysis alert, or in a delayed manner as part of the etiological workup.

Radiological analysis

CT examinations were performed on two multidetector CT scanners available at our institution: a 16-slice Siemens scanner (Siemens Healthineers, Erlangen, Germany) and a 64-slice General Electric scanner (GE HealthCare, Chicago, IL, USA), the choice depending on availability at the time of admission. All CTAs were independently reviewed by the same senior neuroradiologist to confirm the presence of aortic thrombus and to rule out differential diagnoses, including unstable aortic plaque and aortic angiosarcoma. Aortic thrombus was defined as an intraluminal filling defect with well-defined margins, clearly distinct from the aortic wall and visualized within the aortic lumen on at least two imaging planes, without evidence of attachment to the vessel wall. In patients with available follow-up CT angiography, complete disappearance of the lesion on follow-up imaging further supported the diagnosis of thrombus. These features allowed differentiation from complex or ulcerated atherosclerotic plaque. For each thrombus, the location, the maximal dimensions, and the extension to the supra-aortic branches were recorded, together with the condition of the underlying aortic wall (normal, atheromatous, or aneurysmal). The systematic review of all examinations by a single experienced reader ensured consistency of the radiological assessment across patients, irrespective of the scanner used. Follow-up CTA was initially planned at approximately 10 days after the diagnosis of aortic thrombus, although the timing could vary depending on imaging availability. Thrombus resolution was assessed by comparing the follow-up CTA with the baseline examination, including the dimensions and extent of the thrombus. Complete resolution was defined as complete disappearance of the previously identified intraluminal filling defect. Partial resolution was defined as a persistent but reduced thrombus compared with baseline. When a residual lesion was observed, a subsequent follow-up CTA was performed when possible to confirm its persistence and to differentiate residual thrombus from an underlying atherosclerotic plaque.

Variables

In our study, we assessed sociodemographic factors (age and sex), medical history (particularly cardiovascular risk factors, infectious diseases, and systemic diseases), clinical data (chief complaint, time to presentation, and initial clinical examination), radiological data (non-contrast CT scan, initial CTA, and follow-up CTA after treatment), and outcome. Neurological outcomes were assessed using the National Institutes of Health Stroke Scale (NIHSS) and the modified Rankin Scale (mRS) [7, 8].

Data collection

Study data were collected using a pre-established data collection form based on patients' electronic medical records (SIH HOSIX). Patients were hospitalized in our department to initiate therapeutic anticoagulation, ensure close clinical follow-up, and complete the etiological workup for the aortic thrombus. A follow-up CTA was subsequently performed, the results of which guided further therapeutic management.

Aim of the study

The primary objective was to describe aortic thrombus as a potential cause of ischemic stroke and to characterize the clinical, radiological, and therapeutic features of the patients. The use of DOACs was also reported descriptively as part of our experience, without attempting to assess treatment efficacy

Statistical analysis

Data were recorded and analyzed using Microsoft Excel 2010 (Microsoft Corp., Redmond, WA, USA). Descriptive statistics were used to summarize the data

Ethical considerations

An anonymous database was created from patients' medical records, accessible exclusively within our department. No information that could lead to patient identification was included.

## Results

This study included eight patients, comprising six females (75%) and two males (25%), with a mean age of 65.87 years (range: 50-78). At the end of the study period, one patient had died, and two were lost to follow-up. Regarding cardiovascular risk factors, three patients had hypertension (37.5%), and one had diabetes mellitus (12.5%). Four patients had no known modifiable cardiovascular risk factors. One female patient, aged fifty, presented with systemic manifestations suggesting a vasculitic etiology of the aortic thrombus. Apart from the cerebral location, no peripheral embolic event was recorded, either before the ischemic stroke or after initiation of therapeutic anticoagulation. The mean time to presentation to the emergency department of Hassan II University Hospital was 12 hours and 56 minutes, with a range of three to 48 hours. Six patients presented within nine hours of neurological deficit onset. The NIHSS score at admission ranged from 0 to 22, with a mean of 6.

**Figure 1:**
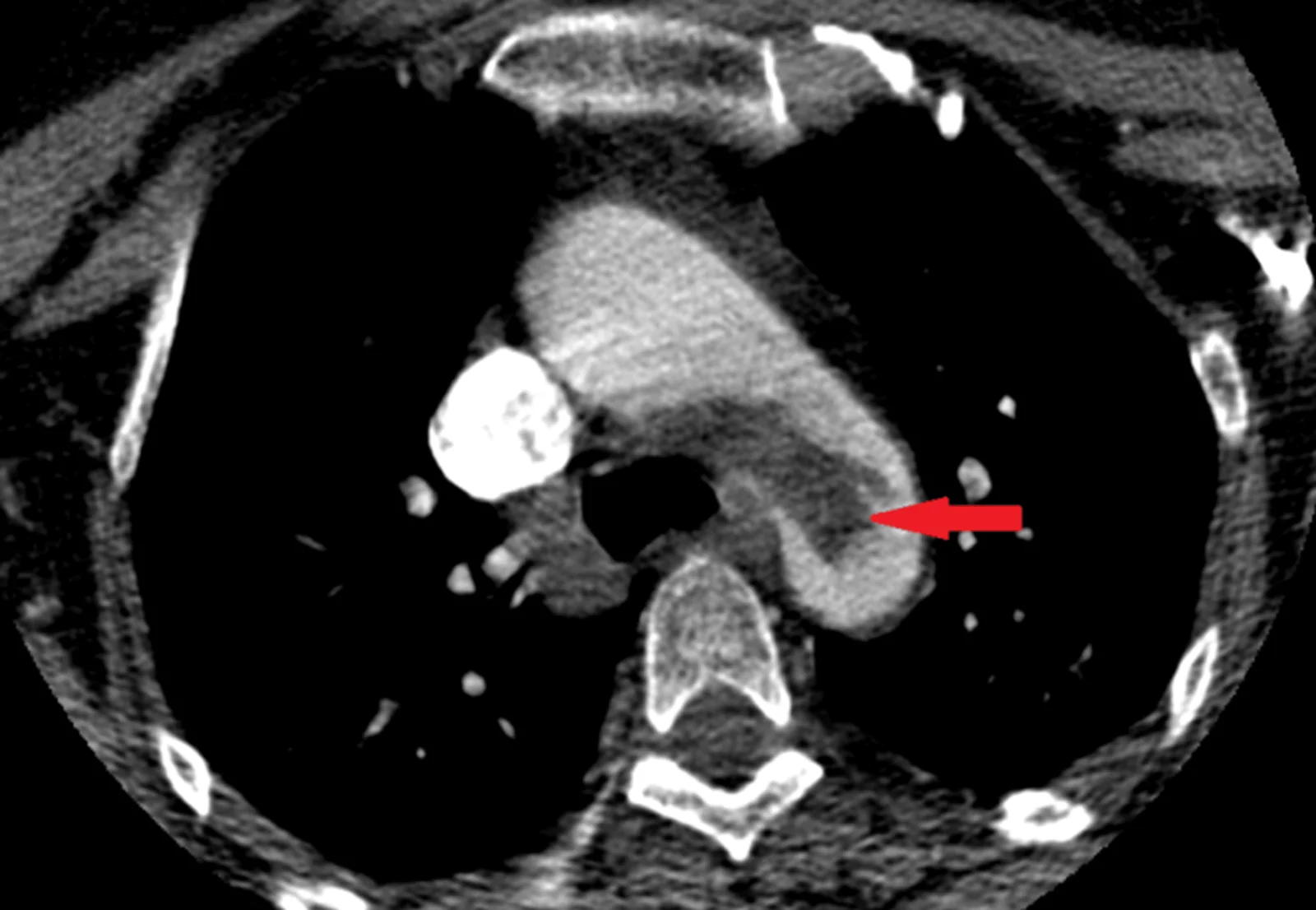
CTA showing a floating thrombus of the aortic arch in a 58-year-old woman (Case 5)

**Figure 2:**
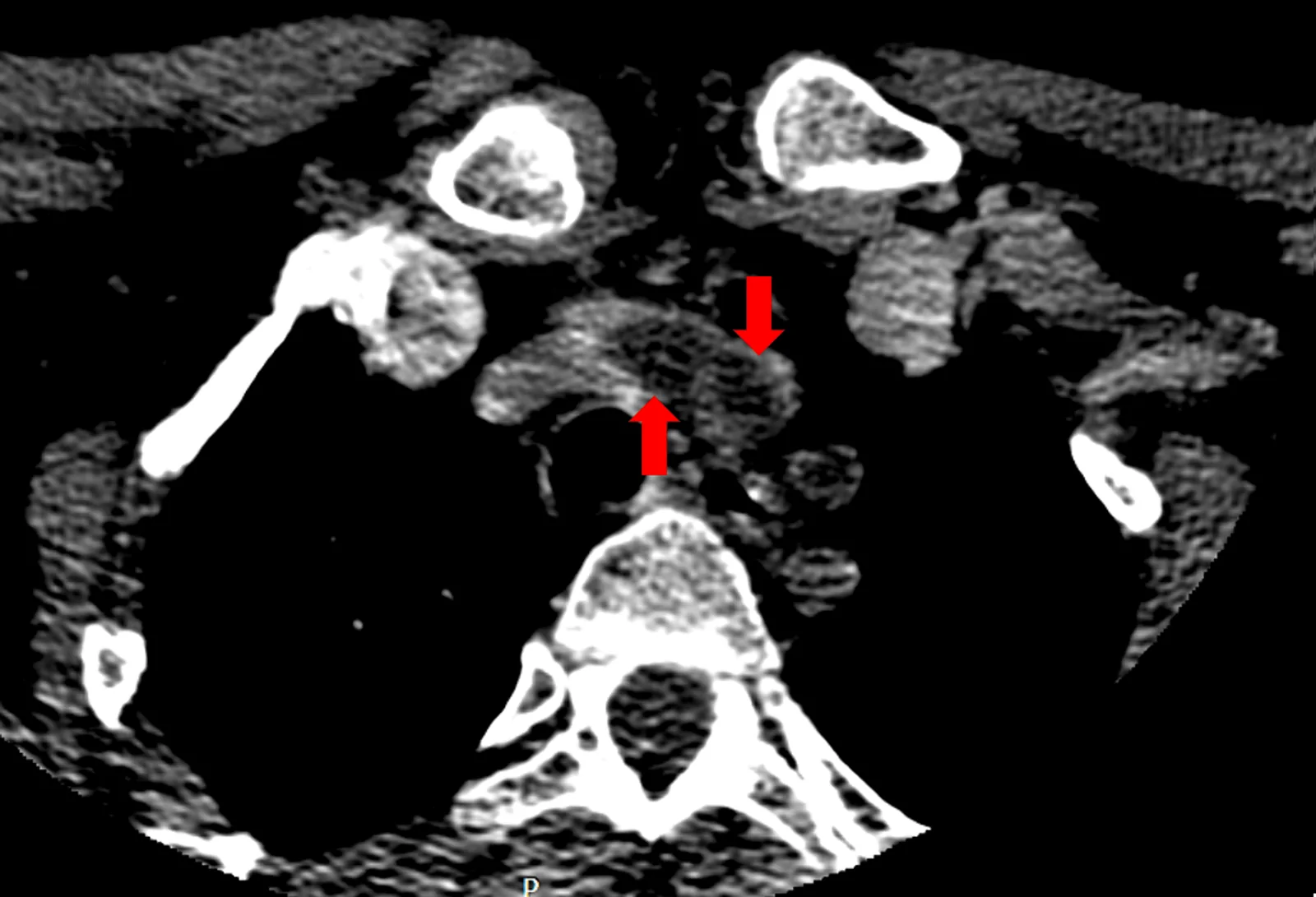
CTA showing a floating aortic thrombus extending to the brachiocephalic arterial trunk and the left common carotid artery in a 50-year-old female patient (Case 2)

Initial non-contrast CT (NCCT) scan revealed subacute ischemic lesions in three patients: two in the middle cerebral artery (MCA) territory and one in the posterior inferior cerebellar artery (PICA) territory. In three additional patients, no obvious hypodensity was identified on the initial NCCT scan, with an ASPECTS of eight in two patients and six in one patient. The remaining two patients had no evidence of acute ischemic lesions on initial imaging, with an ASPECTS of 10. In these five patients without an obvious acute hypodensity on initial imaging, subsequent follow-up NCCT scans demonstrated superficial MCA territory ischemic lesions. Regarding CTA findings, the aortic arch was the predominant site of thrombus, identified in seven patients (Figure 1). One patient had a thrombus located in the descending aorta. The thrombus extended into the three supra-aortic vessels in two patients, and into the brachiocephalic artery only in two other patients (Figure 2).

Unstable atheromatous aortic plaque was the main etiology of aortic thrombus in our study, identified in six patients. One patient had an aneurysm of the ascending aorta. A systemic etiology was suspected in one patient but could not be confirmed, as she died before the completion of the etiological workup. On CTA, no abnormality of the circle of Willis was identified in four patients; one patient had a middle cerebral artery thrombus at the M1 portion, and three patients had non-occlusive atheromatous plaques. Table 1 summarizes CTA data in our series.

**Table 1:** CTA data in our series ᵃ One of these patients also presented with aneurysmal dilatation of the aorta

Radiological parameter	Finding	Patients, n
Thrombus location	Aortic arch	7
	The descending aorta	1
Thrombus extent	Three supra-aortic vessels	2
	Brachiocephalic artery	2
Status of underlying aorta	Unstable atheromatous plaque	6ᵃ
	Normal	2
Status of Willis circle	Normal	4
	Non-occlusive atheromatous plaques	3
	Thrombus	1

Transesophageal echocardiography was performed in one patient but failed to visualize the aortic thrombus, which was ultimately diagnosed on CTA. A complete etiological work-up for ischemic stroke, including transthoracic echocardiography, electrocardiography, and evaluation of the supra-aortic trunks, did not identify any alternative cause in any of the patients. In this context, the presence of an aortic thrombus was considered the most likely etiological explanation for the ischemic stroke, although a definitive causal relationship cannot be established in every patient.

Regarding therapeutic management, intravenous thrombolysis and mechanical thrombectomy were not performed in any patient in our series, due to the risk of inducing peripheral embolism. All patients were started on therapeutic anticoagulation with low-molecular-weight heparin (LMWH), specifically enoxaparin (Lovenox®), administered subcutaneously at a dose of 100 IU anti-Xa/kg (1 mg/kg) every 12 hours for a mean duration of 10 days, in combination with antiplatelet therapy (aspirin 100 mg daily). Subsequently, in patients with persistent thrombus on follow-up imaging, enoxaparin was either continued or switched to oral anticoagulation (DOAC) with rivaroxaban at a dose of 20 mg per day until thrombus resolution.

**Figure 3:**
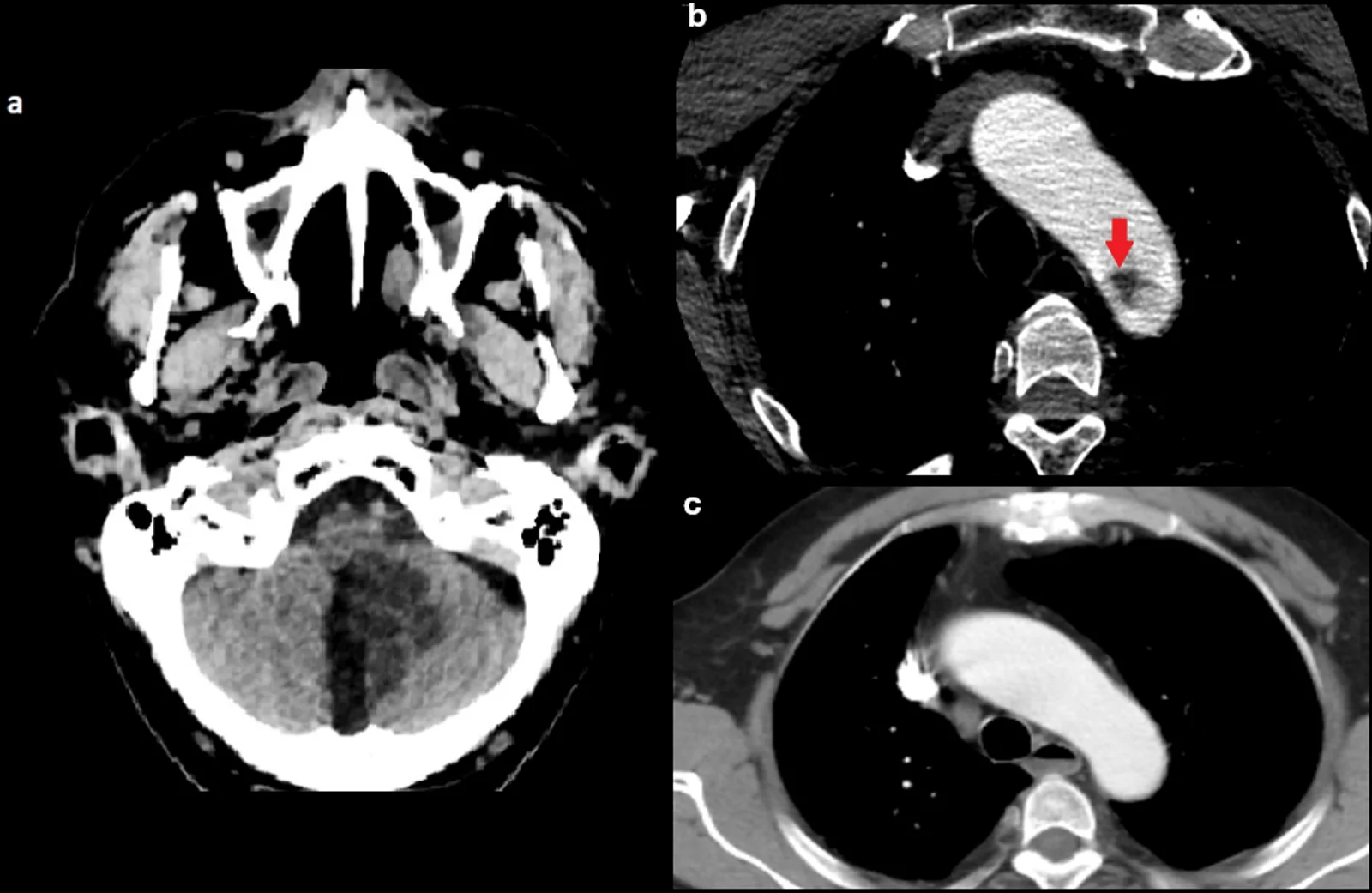
(a) CT scan image of a 63-year-old patient with an ischemic stroke in the left PICA territory, (b) CT scan image showing a floating thrombus of the aortic arch in the same patient, (c) follow-up CT scan image demonstrating complete thrombus resolution after 9 days of therapeutic anticoagulation with low molecular weight heparin (Case 6)

Follow-up CTA was performed at a mean of eleven days after treatment initiation and showed complete thrombus resolution in two patients (Figure 3), partial resolution in three patients, and persistence of the thrombus in two patients. One patient did not undergo follow-up CTA and was started on oral anticoagulation due to poor therapeutic adherence.

A second follow-up CTA was performed in three patients at a mean of nineteen days after treatment initiation, showing complete thrombus resolution in all three. One patient was lost to follow-up and did not undergo further imaging, and one died before the second follow-up CTA could be performed. One patient who was started on a DOAC developed a gastrointestinal hemorrhage. The mean duration of therapeutic anticoagulation to achieve complete thrombus resolution was 28 days.

The mean duration of hospitalization was 11 days (range: seven to 24 days). One patient died following transfer to the ICU due to clinical and radiological deterioration (massive ischemic stroke with mass effect). The mean NIHSS score upon discharge of the seven remaining patients was 6, with a mean mRS of 2. Table 2 summarizes the demographic, radiological, and therapeutic data of the patients in our series.

**Table 2:** Demographic, imaging, and management data of patients in our series F: female; M: male; MCA: middle cerebral artery; LMWH: Low-molecular-weight heparin; DAPT: dual antiplatelet therapy; DOAC: direct oral anticoagulant; PICA: posterior inferior cerebellar artery; N/A: not applicable.

Case	Sex, Age (years)	Initial CT findings	Initial management	Follow-up CT findings	Further management
1	F, 66	Non-contrast CT showing no abnormalities. Floating thrombus overlying an unstable aortic arch plaque	Therapeutic anticoagulation with LMWH and aspirin 100 mg daily for 10 days	Right superficial MCA territory infarct. Complete resolution of the aortic thrombus	DAPT (aspirin + clopidogrel) for one month, followed by aspirin 100 mg daily
2	F, 50	Total left MCA territory infarct with mass effect. Floating aortic arch thrombus extending into the brachiocephalic artery, left common carotid artery, and left subclavian artery	Therapeutic anticoagulation with LMWH and aspirin 100 mg daily for 7 days	Total left MCA territory infarct with subarachnoid hemorrhage. Partial resolution of the aortic thrombus	Therapeutic anticoagulation extended to 21 days (LMWH switched to DOAC), then aspirin upon thrombus resolution
3	F, 77	Non-contrast CT showing no abnormalities. Floating aortic arch thrombus extending into the brachiocephalic artery, overlying an aortic arch plaque	Therapeutic anticoagulation with LMWH and aspirin 100 mg daily for 9 days	Left superficial MCA territory infarct. No change in thrombus size	Therapeutic anticoagulation maintained, switching from LMWH to a DOAC. Poor DOAC adherence. Lost to follow-up
4	F, 60	ASPECTS score of 8/10. Floating aortic arch thrombus extending into the brachiocephalic artery, overlying an unstable aortic arch plaque	Therapeutic anticoagulation with LMWH and aspirin 100 mg daily for 12 days	Left superficial MCA territory infarct. Complete resolution of the aortic thrombus	DAPT (aspirin + clopidogrel) for one month, followed by aspirin 100 mg daily
5	F, 58	Deep left MCA territory infarct. Floating aortic arch thrombus extending into the brachiocephalic artery, left common carotid artery, and left subclavian artery	Therapeutic anticoagulation with LMWH and aspirin 100 mg daily for 14 days	Total left MCA territory infarct with mass effect. Partial resolution of the thrombus	Deceased
6	M, 63	Left PICA territory cerebellar infarct. Floating aortic arch thrombus overlying an aortic arch plaque. Aneurysmal dilatation of the ascending thoracic aorta	Therapeutic anticoagulation with LMWH and aspirin 100 mg daily for 5 days	Left PICA territory cerebellar infarct. Partial resolution of the thrombus	Therapeutic anticoagulation with LMWH for 9 days, then DAPT for 1 month upon thrombus resolution, followed by aspirin 100 mg daily
7	M, 75	ASPECTS score of 6/10. Floating descending aortic thrombus overlying an atherosclerotic plaque	Therapeutic anticoagulation with LMWH and aspirin 100 mg daily for 7 days	Left superficial MCA territory infarct. No change in thrombus size	Therapeutic anticoagulation maintained for a total of 1 month, switching from LMWH to DOAC. Follow-up CTA showing thrombus stability; therapeutic anticoagulation continued for a total of 3 months, then switched to aspirin monotherapy upon thrombus resolution
8	F, 78	ASPECTS score of 8/10. Floating aortic arch thrombus overlying an aortic arch plaque	Therapeutic anticoagulation with LMWH and aspirin 100 mg daily for 10 days, then switched to a DOAC due to poor adherence to LMWH	Left superficial MCA territory infarct and right PICA territory cerebellar infarct. Follow-up CTA not performed; patient lost to follow-up	N/A

## Discussion

Aortic thrombus is considered a rare and poorly recognized source of peripheral emboli, particularly ischemic stroke and transient ischemic attack [3]. It is more frequent in the elderly, as reflected in our study, where the mean age was 65.87 years, suggesting that unstable atheromatous aortic plaque remains the most common underlying cause [3]. Aortic thrombus can also occur in younger adults, particularly in the setting of thrombophilia, infectious aortitis such as syphilis, or systemic diseases and vasculitides such as antiphospholipid syndrome [4].

The main underlying cause of aortic thrombus is aortic atherosclerosis, consistent with our study's results, in which ulcerative atheromatous aortic plaques were found in six patients (75%). In the literature, an aortic plaque carries a risk of thrombus formation when its maximal thickness exceeds 4 mm, when ulcerations deeper than 2 mm are present, or when it appears hypoechoic on transesophageal echocardiography [5]. As observed in our study, the most common site of thrombus formation is the transverse thoracic aorta and the isthmus [6].

Thrombus of the descending thoracic aorta can also lead to ischemic stroke through embolization to the supra-aortic trunks via diastolic retrograde aortic flow [9]. This phenomenon most commonly involves the left subclavian artery, less frequently the left common carotid artery, and rarely the brachiocephalic arterial trunk [10]. Harloff's study, using 3D and 4D MRI techniques, showed that this retrograde flow affected the left subclavian artery in 67% of cases, the left common carotid artery in 24.7% of cases, and the brachiocephalic arterial trunk in 14.4%. In that study, cryptogenic ischemic strokes related to diastolic retrograde aortic flow were distributed across cerebral arterial territories: posterior circulation in 6 out of 9 patients, the left cerebral hemisphere in two patients, and the right cerebral hemisphere in 1 patient [11].

Transesophageal echocardiography (TEE) has long been considered the reference tool for the diagnosis of aortic thrombus [12]. However, it fails to visualize the aortic portion obscured by the trachea. CTA and MRA are currently the primary imaging modalities for diagnosing aortic thrombus. MRA can also rule out the main differential diagnosis, namely aortic angiosarcoma. In our series, transesophageal echocardiography could not be performed in the emergency department. The diagnosis of aortic thrombus was established using supra-aortic trunk CTA, which further underlines the importance of including a systematic evaluation of the different aortic segments in imaging protocols for patients presenting with acute-onset neurological deficits, particularly given that the identification of an aortic thrombus would directly impact the therapeutic approach, especially regarding the indication and risks of intravenous thrombolysis.The optimal therapeutic approach for aortic thrombus remains controversial, due to the lack of large prospective series in the literature. Lapreche T and Choukroun EM concluded in their studies, conducted in 1997 and 2002 respectively, that vitamin K antagonists (VKA) were superior to antiplatelet therapy, highlighting the importance of regular long-term monitoring [13, 14]. These authors proposed initiating therapeutic anticoagulation with enoxaparin or calciparin for two weeks, followed by monitoring with TEE. In the event of thrombus resolution or regression, they proposed maintaining VKA therapy for 6 months with an INR between 2 and 2.5, although no clear justification is provided for this duration given that the thrombus had already resolved. If the thrombus remained stable, they proposed thrombectomy with cerebral protection under extracorporeal circulation [13, 14]. Therapeutic anticoagulation combined with antiplatelet therapy is therefore the first-line treatment for aortic thrombus [3]. However, the duration of anticoagulation is not specified by most authors. In our series, anticoagulation was maintained for a minimum of 10 days, while the mean time to complete thrombus resolution was 28 days.

There is no clear consensus in the literature regarding the use of direct oral anticoagulants (DOAC) in the treatment of aortic atherosclerosis and aortic thrombus [15]. They have been used in some reported cases, leading to complete thrombus resolution, with a proposed treatment duration ranging from 3 weeks to one year [16-18]. In our series, DOACs were prescribed in four patients who had initially been treated with enoxaparin at a therapeutic dose, after a first follow-up CTA revealed persistent aortic thrombus. Among these four patients, two underwent a second follow-up CTA, both showing complete thrombus resolution; the remaining two did not undergo further imaging as they were lost to follow-up.

Our series highlights the potential adverse effects of therapeutic anticoagulation, particularly with DOAC, especially given that the population at risk of aortic thrombus is predominantly elderly, carrying an inherently high risk of hemorrhage, especially gastrointestinal bleeding. This further underlines the importance of carefully assessing this risk, particularly when anticoagulant and antiplatelet therapies are combined.

The benefit of thrombolysis remains debated. It is mostly not recommended by most authors due to the risk of thrombus fragmentation [13]. However, it has been successfully used without complications in some reported cases, including one patient with mesenteric infarction secondary to aortic thrombus and another with ischemic stroke [19, 20]. As for mechanical thrombectomy of the aortic thrombus, it is reserved for specific cases such as a highly mobile thrombus with or without recurrent peripheral emboli, or the persistence of residual thrombus despite therapeutic anticoagulation [14]. Many authors have proposed surgery as the first-line treatment, based on a high risk of recurrence under medical treatment, particularly in young adults. This procedure consists of a median sternotomy with cardiopulmonary bypass and circulatory arrest under deep hypothermia, with intraoperative echocardiographic monitoring to assess the location of the aortic thrombus and detect any potential migration, along with resection of the thrombus attachment site to prevent recurrence [21, 22].

Our study has several limitations, including its retrospective nature, the small sample size, and the loss to follow-up of two patients.

## Conclusions

Aortic thrombus is an underrecognized etiology of ischemic stroke, occurring in both elderly and young adults across different clinical settings (atherosclerosis vs. systemic and infectious diseases). While TEE was historically the primary diagnostic tool, MRA and CTA are currently the main imaging modalities for diagnosing the thrombus, assessing its location, characterizing the underlying atheromatous plaque, and ruling out differential diagnoses. The optimal treatment has yet to be codified but relies mostly on therapeutic anticoagulation and correction of cardiovascular risk factors, or treatment of the underlying etiology. Our series highlights the importance of including supra-aortic trunk CTA in the imaging workup of ischemic stroke to identify aortic thrombus, which may represent a potential etiological explanation after exclusion of other possible causes. The presence of an aortic thrombus has been considered a potential contraindication to thrombolysis by some authors, although the available evidence remains limited and treatment decisions should take into account thrombus morphology, mobility, embolic risk, and the individual clinical context. In our limited series, DOACs were used in a subset of patients and may represent a potential therapeutic option; however, given the small sample size and loss to follow-up, these observations should be considered hypothesis-generating and do not allow conclusions regarding treatment efficacy.
